# Effects of Age and Sex on Properties of Lumbar Erector Spinae in Healthy People: Preliminary Results From a Pilot Study

**DOI:** 10.3389/fphys.2021.718068

**Published:** 2021-09-20

**Authors:** Zugui Wu, Yi Wang, Zixuan Ye, Yingxing Guan, Xiangling Ye, Zehua Chen, Congcong Li, Guoqian Chen, Yue Zhu, Jianping Du, Guocai Chen, Wengang Liu, Xuemeng Xu

**Affiliations:** ^1^The Fifth Clinical Medical College, Guangzhou University of Chinese Medicine, Guangzhou, China; ^2^Zhejiang Provincial Hospital of Chinese Medicine, Hangzhou, China; ^3^Baishui Health Center, Qujing, China; ^4^Guangdong Second Traditional Chinese Medicine Hospital, Guangzhou, China; ^5^Foshan Hospital of Traditional Chinese Medicine, Guangzhou University of Chinese Medicine, Guangzhou, China

**Keywords:** muscle tone, stiffness, age, sex, properties, lumbar erector spinae

## Abstract

**Background:** The influences of age and sex on properties of lumbar erector spinae have not been previously studied. Changes in the performance of lumbar erector spinae properties associated with age represent a valuable indicator of risk for lower-back-related disease.

**Objective:** To investigate the lumbar erector spinae properties with regard to age and sex to provide a reference dataset.

**Methods:** We measured muscle tone and stiffness of the lumbar erector spinae (at the L3–4 level) in healthy men and women (50 young people, aged 20–30 years; 50 middle-aged people, aged 40–50 years; and 50 elderly people, aged 65–75 years) using a MyotonPRO device.

**Results:** In general, there are significant differences in muscle tone and stiffness among young, middle-aged, and elderly participants, and there were significant differences in muscle tone and stiffness between men and women, and there was no interaction between age and sex. The muscle tone and stiffness of the elderly participants were significantly higher than those of the middle-aged and young participants (*P* < 0.01), and the muscle tone and stiffness of the middle-aged participants were significantly higher than those of the young participants (*P* < 0.01). In addition, the muscle tone and stiffness of men participants were significantly higher than that of women participants (*P* < 0.01).

**Conclusion:** Our results indicate that muscle tone and stiffness of the lumbar erector spinae increase with age. The muscle tone and stiffness of the lumbar erector spinae in men are significantly higher than in women. The present study highlights the importance of considering age and sex differences when assessing muscle characteristics of healthy people or patients.

## Introduction

Chronic low-back pain (CLBP) is common among middle-aged (i.e., aged 40–59 years) and elderly people (i.e., aged over 65 years). A survey study in many countries found that the total prevalence of chronic low back pain reached 7% (Stubbs et al., 2016), which seriously affected the public health and caused a huge social and economic burden (Freburger et al., 2009). Studies have confirmed that the changes of paravertebral muscles have an essential relationship with the occurrence of chronic low back pain (Hides et al., 1996; Sions et al., 2017). Assessing the properties of paravertebral muscles is of great significance and can provide vital information for clinical diagnosis and treatment of chronic low back pain.

The properties of the muscle are important factors influencing muscle performance. Muscle tone is the tension/viscoelasticity (internal) of the muscle in a state of complete relaxation without voluntary contraction (Schoenrock et al., 2018). Abnormally high muscle tone will hinder the blood supply, which will lead to faster muscle fatigue and slower muscle recovery. Stiffness reflects the ability of a muscle to resist contraction or to resist mechanical forces (external) that deforms the muscle. When the muscle stiffness is abnormally high, it takes more effort to stretch the stiff antagonist muscles, resulting in low exercise efficiency. Muscle stiffness is one of the critical indicators of energy storage of the muscle-tendon unit, which has an important influence on the control of joint movement (Kocur et al., 2019). These properties can change dramatically in the context of disease; for example, patients with Parkinson’s have increased muscle stiffness compared with healthy people of the same age (Marusiak et al., 2010). Thoracolumbar fascia as part of the lumbar back muscle, stiffness of the lumbar extensor myofascial is higher in patients with ankylosing spondylitis than in healthy people of the same age (Andonian et al., 2015). In healthy people, there may also be differences in the properties of muscles. It has been reported that muscle mass and strength decrease with age (Cruz-Jentoft et al., 2010; Rosenberg, 2011), as well as the internal structure of muscle remodeling with age (Uitto, 1986; Junior et al., 2002; Kragstrup et al., 2011), and these factors may cause the properties of muscle to change with age. As we all know, the incidence of musculoskeletal diseases in women is significantly higher than that in men (Agyapong-Badu et al., 2016). The high incidence of musculoskeletal diseases in women may be related to sex differences in muscle strength and muscle stiffness (Taş and Salkin, 2019), and there are sex differences in the types of muscle fibers and muscle fatigue resistance (Hamada et al., 2003; Fröhlich-Zwahlen et al., 2014). There may also be sex differences in the properties of muscles. Because the properties of muscles may change with age and sex, distinguishing the effects of these two variables is important for the diagnosis and treatment of clinical diseases and for related scientific research. So far, no studies have reported whether there are differences in age and sex in the properties of paravertebral muscles.

Studies have shown that the muscle tone of the lower-back and dorsal muscles in the resting state is key in maintaining the stability of the spine (White et al., 2018). With the increasing incidence of CLBP and other low-back diseases in young people (Hu et al., 2018), it is important to test parameters that are associated with these diseases and establish standardized data for evaluating the performance of the lumbar erector spinae, using simple and effective methods. Scientific research of diseases related to the lower back is also highly significant to the field. At present, there are many methods for evaluating the properties of muscle; the choice of methods for use in clinical settings is subjective. The Modified Ashworth Scale (MAS; Bohannon and Smith, 1987) and Tardieu Scale (Tardieu et al., 1954) can be used to assess muscle tone, but the reliability has been questioned (Yam and Leung, 2006; Fleuren et al., 2010). Furthermore, the use of these scales is only applicable to the limbs; no relevant scales are available for the clinical evaluation of muscle stiffness. Ultrasound elastography and magnetic resonance elastography can be used to evaluate the properties of muscle (Dresner et al., 2001; Qiu et al., 2015); however, their use in the clinic is limited because of the high cost involved and inconvenience in carrying equipment. Thus, there is a need to develop a simple, rapid, objective, reliable, and low-cost technique for measuring these properties in clinical settings.

The MyotonPRO is an instrument that can measure the properties of muscles. It has many advantages in clinical applications; for example, it is easy to carry and tests can be performed rapidly, with a relatively low cost (Bailey et al., 2013). Current studies have suggested that Myoton technology has utility in diagnosing disease and monitoring changes in the properties of the lower-back and dorsal muscles in the context of lower-back-related diseases (Andonian et al., 2015; White et al., 2018).

The present study aimed to evaluate muscle tone and stiffness of the lumbar erector spinae of participants and to analyze the differences between data from individuals of different ages and sex. The purpose of this was to establish preliminary standardized data for future reference. We hypothesize that muscle tone and stiffness will increase with increasing age, and there are differences in muscle tone and stiffness between men and women. We found that muscle tone and stiffness of the lumbar erector spinae increase with age, and muscle tone and stiffness of the lumbar erector spinae were higher in men than in women. The present study thus achieved the aim of evaluating the utility of the MyotonPRO for these measurements, and has produced a preliminary reference dataset.

## Materials and Methods

### Participants

Through a careful evaluation of the medical history and a physical examination, 150 volunteers who were confirmed as healthy were recruited and divided into six groups based on age and sex (25 elderly men and 25 elderly women, aged 65–75 years, recruited from nearby communities; 25 middle-aged men and 25 middle-aged women, aged 40–50 years, recruited from nearby communities; and 25 young men and 25 young women, aged 20–30 years, recruited from postgraduate students and undergraduate interns in our hospital). Recruitment and data collection was conducted from June 1, 2019 to April 30, 2021. A questionnaire was developed prior to the study, and a member of the research team collected basic information about the participants, including age, sex, height, weight, etc., which was used to screen for recruitment and exclusion criteria. Eligibility was defined by the following inclusion criteria: (Stubbs et al., 2016) no chronic low-back pain (CLBP) or other diseases related to the lower back, and (Freburger et al., 2009) BMI < 30 kg/m^2^. Exclusion criteria were as follows: (Stubbs et al., 2016) spinal disease, such as a spinal tumor, spinal tuberculosis, or other lower-back-related diseases; (Freburger et al., 2009) osteoporosis or other medical issues of the bone; (Sions et al., 2017) neurological disease, such as Parkinson’s disease; (Hides et al., 1996) history of low-back surgery; and (Schoenrock et al., 2018) skeletal-muscle-relaxant drug use.

### Measurement of the Properties of the Lumbar Erector Spinae

**FIGURE 1:**
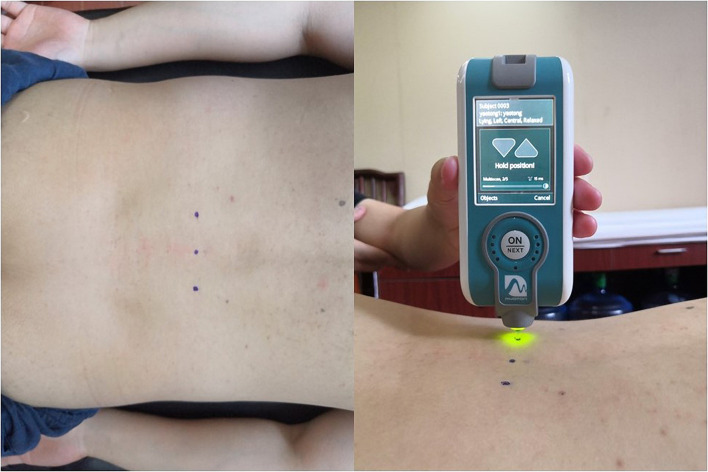
Measurement points and MyotonPRO probe contact measurement points.

MyotonPRO analysis was conducted by a research team member who received 8 h of training from a senior trainer of the Myoton Company. Training included on-site environmental assessments, operation standards and precautions of MyotonPRO, and supervised performance of its operation. Researchers received training over 2 weeks and were familiarized with the operation of MyotonPRO. Before participants were tested, general information, including name, age, height, and weight, was collected. To ensure a quiet test environment, participants rested in bed in a special evaluation room of the hospital for 10 min before the test. Before the start of the measurement, palpate the highest points of the iliac spine on both sides, determine the gap between the L3 and L4 spinous processes, and then mark the measurement point at the most uplift of the lumbar erector spinae on both sides of this gap. The postures of all participants were the same; participants were in the prone position during the test, with the head in a neutral position (the head was placed on a support with a breathing hole) and the upper limbs placed along both sides of the body. Before the MyotonPRO measurement, the surface EMG (sEMG) electrode sheet was attached to the distance of 2.5 cm from the measuring point. Before the MyotonPRO probe touched the skin, the electrode sheet was removed, and then the MyotonPRO probe touched the skin for measurement. Finally, the electrode sheet was applied again to avoid the interaction between the electrode sheet and MyotonPRO (Nair et al., 2016). The muscle state was monitored using sEMG, and the root mean square of the sEMG signal at rest was confirmed as less than 5 μV. The operator held the MyotonPRO in one hand, and placed the device probe vertically at the marking point, then pressed the probe to the required depth. The lamp changed from red to green when the probe had reached the required depth. The instrument provides a constant pre-pressure of 0.18 N + a force of 0.4 N = 0.58 N, lasting for 15 ms, which induces the tissue under the probe to produce a natural damping oscillation. Based on the oscillation signal, the MyotonPRO device automatically calculated the oscillation frequency (Hz) and dynamic stiffness (N/m). The damped oscillation frequency (F; Hz) represents muscle tone in the resting state, that is, the tension/viscoelasticity (internal) of the muscle in the state of no spontaneous contraction and complete relaxation. A higher damped oscillation frequency indicates higher muscle tone (Gavronski et al., 2007; Bailey et al., 2013; Schneider et al., 2015). The dynamic stiffness (S; N/m) represents muscle stiffness, which reflects the ability of the muscle to resist contraction or to resist mechanical forces (external) that deforms the muscle. Higher dynamic stiffness reflects harder muscle (Gavronski et al., 2007; Schneider et al., 2015). Participants were asked to hold their breath for 5 s at the end of an exhalation to reduce the influence of changes in the contact surface. After the test, the coefficient of variation (CV) was evaluated. If the CV was more than 3%, the test was repeated (Mullix et al., 2012). The average values of measurement results of left and right lumbar erector spinae were calculated and used for statistical analysis. The location of the measurement points at the waist of the participants and the MyotonPRO probe contact measurement points were shown in Figure 1.

### Statistical Analysis

Statistical analyses were performed using SPSS 26.0 software (IBM, Armonk, NY, United States). By descriptive statistics, data were summarized as the mean and standard deviation. A two-way analysis of variance was used to analyze the effects of age and sex on the muscle tone and stiffness of the lumbar erector spinae. If there is a statistically significant difference, a *post hoc* analysis will be performed. Sex differences within each age group were statistically analyzed using the Paired-Samples *T*-Test. Meanwhile, analysis of variance was used to determine whether there is an interaction between age and sex. The level of significance was set at *P* < 0.05.

## Results

### Study Population

Baseline characteristics of the study population are shown in Table 1.

**TABLE 1:** Baseline condition of subjects (*n* = 150).

	Young		Middle-aged		Elderly	
	Males (*n* = 25)	Females (*n* = 25)	Males (*n* = 25)	Females (*n* = 25)	Males (*n* = 25)	Females (*n* = 25)
Age (years)	25.44 ± 1.41	24.92 ± 1.52	46.08 ± 3.42	45.24 ± 3.32	68.04 ± 2.70	68.88 ± 3.35
Height (cm)	171.84 ± 5.42	164.92 ± 4.48	168.32 ± 4.85	163.24 ± 4.93	166.76 ± 5.50	159.56 ± 5.58
Weight (kg)	67.68 ± 6.25	61.17 ± 5.09	66.07 ± 9.78	61.28 ± 10.37	64.73 ± 9.10	58.08 ± 9.28
BMI (kg/m^2^)	22.89 ± 1.44	22.46 ± 1.22	23.22 ± 2.61	22.88 ± 2.86	23.22 ± 2.60	22.74 ± 2.98

*Data are presented as mean ± standard deviation. BMI, body mass index.*

### Statistical Results of the Properties of Lumbar Erector Spinae

**FIGURE 2:**
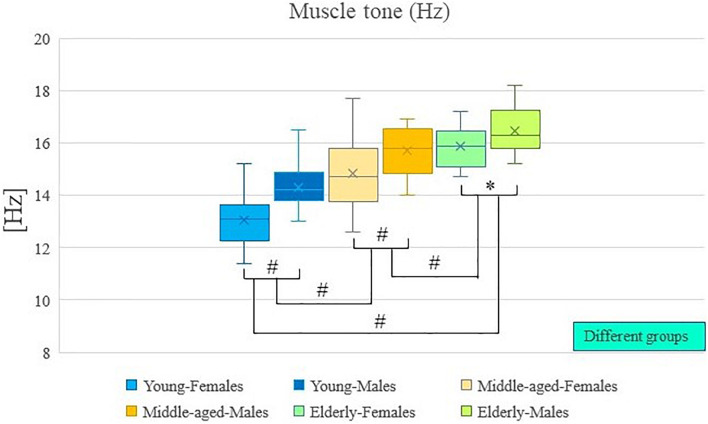
Age and sex differences in muscle tone. ^#^Indicates *P* value < 0.01; ^∗^Indicates *P* value < 0.05.

**FIGURE 3:**
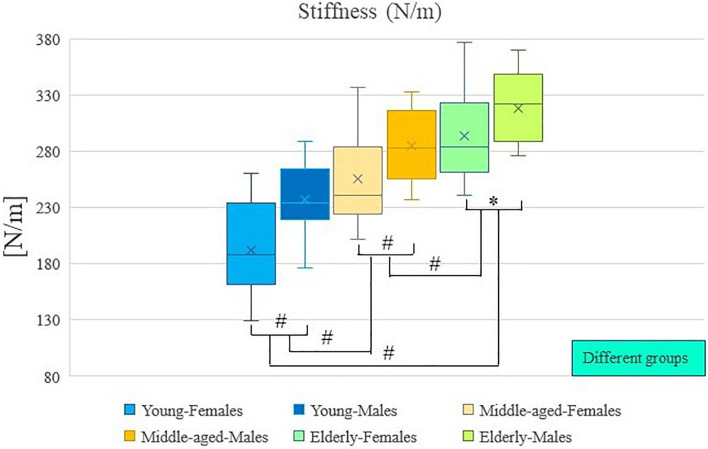
Age and sex differences in stiffness. ^#^Indicates *P* value < 0.01; ^∗^Indicates *P* value < 0.05.

The results of descriptive statistics were shown in Table 2. Table 2 shows the mean and standard deviation of muscle tone and stiffness for each group of participants (by age and sex), as well as the range of these results. Two-way analysis of variance showed that there were significant differences in muscle tone and stiffness between participants of different ages (*P* < 0.01; Table 3), and there were significant differences in muscle tone and stiffness between male and female participants (*P* < 0.01; Table 3). In Figures 2, 3, the two groups were connected by lines to indicate that the comparison between the two groups was carried out. The symbols of statistical differences were marked on the connection lines between the two groups. The distribution and difference of muscle tone and stiffness in different age and sex groups were shown in Figures 2, 3.

**TABLE 2:** Properties of the lumbar erector spinae.

Age	Sex		Muscle tone	Stiffness
Young group	Males	Mean ± SD	14.28 ± 0.78	236.68 ± 30.90
		Range	13.00−16.50	176.00−289.00
	Females	Mean ± SD	13.04 ± 0.99	191.80 ± 37.48
		Range	11.40−15.20	129.50−260.00
Middle-aged group	Males	Mean ± SD	15.72 ± 0.87	285.34 ± 30.33
		Range	14.00−16.90	236.50−333.00
	Females	Mean ± SD	14.82 ± 1.40	255.34 ± 36.89
		Range	12.60−17.70	201.50−337.00
Elderly group	Males	Mean ± SD	16.45 ± 0.86	318.42 ± 30.75
		Range	15.20−18.20	276.00−370.50
	Females	Mean ± SD	15.87 ± 0.75	294.02 ± 37.46
		Range	14.70−17.20	241.00−377.00

**TABLE 3:** Statistical analysis results of main effects and interaction effects.

Factors	Properties	*F*	*P* values
Sex	Muscle tone	32.47	< 0.001^#^
	Stiffness	35.25	< 0.001^#^
Age	Muscle tone	84.82	< 0.001^#^
	Stiffness	92.23	< 0.001^#^
Sex*Age	Muscle tone	1.42	0.245*
	Stiffness	1.20	0.304*

*Indicates Statistical Analysis of Interaction effects. ^#^Indicates *P* value < 0.01.

*Post hoc* analysis showed that the muscle tone and stiffness among elderly participants were significantly higher compared with those of middle-aged participants (*P* < 0.01; Table 4). *Post hoc* analysis showed that the muscle tone and stiffness among elderly participants were significantly higher compared with those of young participants (*P* < 0.01; Table 4). *Post hoc* analysis showed that the muscle tone and stiffness of middle-aged participants were significantly higher compared with those of young participants (*P* < 0.01; Table 4).

**TABLE 4:** Statistical results of *post hoc* analysis.

Properties	Age	Mean difference	95% CI		*P* values
			Lower	Upper	
Muscle tone	YG vs. MG	–1.61	–1.99	–1.22	<0.001^#^
	YG vs. EG	–2.50	–2.88	–2.11	<0.001^#^
	MG vs. EG	–0.89	–1.27	–0.50	<0.001^#^
Stiffness	YG vs. MG	–56.10	–69.59	–42.60	<0.001^#^
	YG vs. EG	–91.98	–105.47	–78.48	<0.001^#^
	MG vs. EG	–35.88	–49.37	–22.38	<0.001^#^

*YG, young group; MG, middle-aged group; EG, elderly group. ^#^Indicates *P* value < 0.01.*

Analysis of variance showed that there were significant differences in muscle tone and stiffness between male and female participants (*P* < 0.01; Table 4). The muscle tone and stiffness of the lumbar erector spinae of male participants were significantly higher than those of females. The Paired Sample *T*-test showed that in the young group, middle-aged group, and elderly group, there were sex differences in muscle tone and stiffness within the group. The muscle tone and stiffness of the lumbar erector spinae of male participants were significantly higher than those of females (*P* < 0.05; Table 5).

**TABLE 5:** Statistical results of paired-samples *T* Test.

Properties	Sex	SEM	95% CI		*P* values
			Lower	Upper	
Muscle tone	Young group	0.26	–1.78	–0.68	< 0.001^#^
	Middle-aged group	0.30	–1.54	–0.27	0.007^#^
	Elderly group	0.22	–1.04	–0.10	0.019*
Stiffness	Young group	10.40	–66.35	–23.40	< 0.001^#^
	Middle-aged group	9.05	–48.68	–11.31	0.003^#^
	Elderly group	9.84	–44.72	–4.07	0.021*

^#^Indicates *P* value < 0.01. *Indicates *P* value < 0.05.

A two-way analysis of variance showed that there was no significant difference in the interaction between age and sex on muscle tone (*P* > 0.05; Table 3). A two-way analysis of variance showed that there was no significant difference in the interaction between age and sex on stiffness (*P* > 0.05; Table 3).

## Discussion

In the present study, the effects of age and sex on muscle tone and stiffness of the lumbar erector spinae were assessed with the MyotonPRO device. We identified age- and sex-related differences in these parameters, and our data of young, middle-aged and elderly healthy men and women supports the hypothesis that age and sex influence muscle tone and stiffness of the lumbar erector spinae.

### Effect of Aging on the Properties of the Lumbar Erector Spinae

Our results of the impact of aging on the lumbar erector spinae are consistent with those of previous studies that used laboratory techniques to investigate the stiffness of the plantar tissue (Blanpied and Smidt, 1993) and ultrasound elastography to assess the stiffness of the biceps (Eby et al., 2015) in relation to aging. In addition, a study used MyotonPRO to evaluate the muscle tone and stiffness of the rectus femoris and biceps brachii muscles. It also found that the muscle tone and stiffness of participants of different ages were significantly different, and the differences between different muscles were inconsistent (Agyapong-Badu et al., 2016). There are many factors that affect the properties of the lumbar erector spinae, and sex and age have significant effects on the properties of the lumbar erector spinae. However, the specific mechanism of the changes in the properties of the lumbar erector spinae is currently unclear.

The properties of muscles and tendons will change with age (Blanpied and Smidt, 1993; Scaglioni et al., 2003). Studies have found that the quality and strength of skeletal muscle will decrease with age (Cruz-Jentoft et al., 2010; Rosenberg, 2011). When the muscle mass decreases to a certain level, the muscle content will decrease, and sarcopenia may even appear (Baumgartner and Waters, 2006), which may affect the properties of the muscle. With aging, the internal structure of the skin, myofascial, and muscle tissue is reshaped (Uitto, 1986; Junior et al., 2002; Kragstrup et al., 2011), and the cells and extracellular matrix of soft tissues such as muscle undergo changes, including a re-adjustment of muscle fiber type, changes in the protein concentration of connective tissue, and structural degeneration of connective tissue (Barros et al., 2002; Haus et al., 2007; Trappe, 2009). The shape of the extracellular matrix, including the content of collagen and elastin, will also affect the properties of muscle stiffness (Purslow, 2002). Therefore, some studies believe that the infiltration and change of connective tissue may be one of the reasons why muscle tone and stiffness increase with age (Kocur et al., 2019). In addition, the deterioration of connective tissue with age may also lead to an increase in muscle stiffness and a decrease in elasticity (Alnaqeeb et al., 1984; Rodrigues and Rodrigues Junior, 2000; Barros et al., 2002). Other studies have found that with age, the number and quality of muscle fibers will change, which is also one of the important reasons that affect the properties of muscles (Mutungi and Ranatunga, 1996; Gosselin et al., 1998; Cvetko et al., 2012). Fast-type muscle fibers (Type II muscle fibers) have higher intrinsic strength and higher stiffness than slow-type muscle fibers (Type I muscle fibers; Purslow, 2002), while slow-type muscle fibers have better durability (Wüst et al., 2008). With age, fast-type muscle fibers will be lost preferentially (Manta et al., 1995), while slow-type muscle fibers change more slowly (Kararizou et al., 2009). The ratio of muscle fibers changes from fast-type fibers to slow-type muscle fibers, which may lead to an increase in stiffness and a decrease in elasticity (Cvetko et al., 2012; Meznaric et al., 2018). In addition, aging is related to the relative increase in the ratio of non-contracting and contracting components in muscle tissue, which may also lead to changes in muscles’ properties (Alnaqeeb et al., 1984).

Previous studies on lumbar erector spinae have revealed to some extent the mechanism by which the properties of lumbar erector spinae change with age (Alnaqeeb et al., 1984; Uitto, 1986; Mutungi and Ranatunga, 1996; Gosselin et al., 1998; Rodrigues and Rodrigues Junior, 2000; Barros et al., 2002; Junior et al., 2002; Purslow, 2002; Baumgartner and Waters, 2006; Haus et al., 2007; Trappe, 2009; Cruz-Jentoft et al., 2010; Kragstrup et al., 2011; Rosenberg, 2011; Cvetko et al., 2012; Meznaric et al., 2018; Kocur et al., 2019). The decrease of muscle mass, the decrease of muscle cross-sectional area, the degeneration of connective tissue, the changes in the quantity and mass of muscle fibers, the adjustment of muscle fiber type, and the remodeling of muscles’ internal structure may be the reasons for the changes in the properties of lumbar erector spinae with increasing age.

### Differences in the Properties of the Lumbar Erector Spinae Between Sex

A study on the rectus femoris and biceps brachii muscles found the muscle tone and stiffness of the muscles to be higher in men than in women (Agyapong-Badu et al., 2016). Previous studies on muscle stiffness around the knee have reported the muscle stiffness of men to be greater than that of women (Wang et al., 2017). Another study examining sex differences in passive gastrocnemius stiffness using ultrasound technology found that stiffness of the internal end of the passive gastrocnemius muscle was greater in men than in women, which further supports the existence of sex differences in the biochemical parameters of muscles (Morse, 2011). Furthermore, A study of patients with Achilles tendon disease has concluded that sex may affect the properties of the Achilles tendon (Morgan et al., 2018). We observed differences in muscle tone and stiffness of the lumbar erector spinae between men and women, which are consistent with previously reported differences in muscle properties between men and women, and this difference may be related to sex differences in muscles’ properties.

Female skeletal muscle exhibits higher anti-fatigue capacity than males, which may be correlated with the proportions of muscle fiber types (Fröhlich-Zwahlen et al., 2014). Some researchers have speculated that female muscles contain more type I muscle fibers than do male muscles, meaning that the slow contraction of type I muscle fibers would confer a strong anti-fatigue capacity to female skeletal muscle (Hamada et al., 2003). In addition, hormone levels *in vivo* have been found to be correlated with muscle stiffness, with estrogen levels in women being negatively correlated with muscle stiffness (Bell et al., 2012). Additionally, the higher percentage of fat tissue in women than in men (Otsuka et al., 2018), and the sex difference in the muscle tone and stiffness of the rectus femoris between men and women may be related to the thickness of subcutaneous fat, and the thickness of subcutaneous fat varies with sex (Agyapong-Badu et al., 2016). The muscle mass and strength of men are higher than that of women (Narici and Maffulli, 2010), and in different age groups and different tissues, women’s muscle volume is smaller than men’s (Banks, 2006; Moore et al., 2014). Muscles stiffness is influenced by factors such as muscles’ cross-sectional area and muscle mass (Narici et al., 1992; Fouré et al., 2012), as well as intrinsic characteristics such as actin-myosin cross-bridge and protein titin (Proske and Morgan, 1999), and the sex difference in hardness may be attributable to the fact that men have a larger cross-sectional area of muscle, better muscle mass, and therefore more actin-myosin cross-bridge and protein titin in the muscle than women (Wang et al., 2015). Males have larger and more skeletal muscle than females, and some muscles have a greater proportion of fast-type muscle fibers (Staron et al., 2000; Porter et al., 2002; Roepstorff et al., 2006), possibly due to sex differences in human skeletal muscle gene expression and the interaction of sex-specific hormones (Welle et al., 2008; Maher et al., 2009; Liu et al., 2010).

Previous studies on lumbar erector spinae have to some extent revealed the mechanism of sex differences in the properties of lumbar erector spinae (Narici et al., 1992; Proske and Morgan, 1999; Staron et al., 2000; Porter et al., 2002; Hamada et al., 2003; Banks, 2006; Roepstorff et al., 2006; Welle et al., 2008; Maher et al., 2009; Liu et al., 2010; Narici and Maffulli, 2010; Bell et al., 2012; Fouré et al., 2012; Fröhlich-Zwahlen et al., 2014; Moore et al., 2014; Wang et al., 2015; Otsuka et al., 2018). The anti-fatigue ability of skeletal muscle, the proportion of different muscle fiber types, the estrogen level in the body, the proportion of adipose tissue, the thickness of subcutaneous fat, the volume and cross-sectional area of muscle may be the reasons for the sex differences in the properties of lumbar erector spinae. Although previous studies have explored the effects of aging and sex on muscles’ properties, the specific mechanism still needs to be further clarified. Further laboratory research is needed to investigate these mechanisms.

## Limitations

This cross-sectional study has several limitations. No other analyses, such as ultrasound elastography and magnetic resonance elastography, were performed simultaneously to allow a direct comparison with the results obtained from the MyotonPRO. However, we have since purchased a muscle-bone ultrasound device, and future studies will involve the combination of ultrasound elastography and MyotonPRO technology. In the present study, the anterior and lateral bending angles of the lower-back spine were not strictly controlled during the test, which may have affected the properties of the lumbar erector spinae. Before the test, exercise levels were not controlled, which may also have affected lumbar erector spinae’s properties. Furthermore, three discrete, non-continuous age groups (20–30, 40–50, and 65–75 years) were included in the present study to represent young, middle-aged, and elderly people (i.e., a restricted range within the standard age group definitions was used), rather than evaluating age as a continuous variable, which would enable a more precise analysis of the influence of age on parameters of the lumbar erector spinae. This study was a preliminary study, and future studies will include participants from a more continuous age range further to investigate the properties of the lumbar erector spinae. Finally, the study included only healthy people for analysis and did not include patients with CLBP as a reference; Considering the complexity of CLBP, there are many influencing factors, and this part of the study will be carried out independently in the future. Although the sample size in this research is sufficient for demonstrating the purpose of this study, further studies with larger sample sizes are required to support the preliminary conclusions of this research.

## Future Research Directions

Further studies in different muscles are required to establish the influence of age and sex on parameters of muscle, as well as the range and standard values of each age group. Research on muscle combining ultrasonic-elastography technology would provide valuable information and support for the present results. Meanwhile, future studies should include more patients with related diseases as control groups to explore the relationship between physiology and pathology further. Currently, the use of MyotonPRO technology is increasing for the detection of various muscle-related diseases, such as Parkinson’s disease, CLBP, ankylosing spondylitis, and so on (Marusiak et al., 2010; Hu et al., 2018; White et al., 2018). Detection of muscle-related diseases is highly important, and attention to the influence of age and sex on the properties parameters of skeletal muscle could be beneficial in this context.

## Conclusion

From our results, we can make the following preliminary conclusions. The effect of aging on the properties of lumbar erector spinae recorded by MyotonPRO equipment shows that muscle tone and stiffness increase with age. Compared with middle-aged people and young people, the muscle tone and stiffness of the lumbar erector spinae of the elderly people are higher; compared with the young people, the muscle tone and stiffness of the lumbar erector spinae of the middle-aged people are higher. Properties of the lumbar erector spinae differ between sex. Muscle tone and stiffness of the lumbar erector spinae are higher in men than in women. This study provides a reference for muscle tone and stiffness of the lumbar erector spinae in young, middle-aged, and elderly healthy people. It is necessary to continue gathering data of other age groups, and to evaluate age as a continuous variable, to form a more complete reference database.

## Data Availability Statement

The raw data supporting the conclusion of this article will be made available by the corresponding author, without undue reservation.

## Ethics Statement

The studies involving human participants were reviewed and approved by Ethics Committee of Guangdong Second Traditional Chinese Medicine Hospital. The patients/participants provided their written informed consent to participate in this study.

## Author Contributions

ZW, GCC, WL, and XX designed the entire research program. ZW, YW, ZY, and YG collected all of the data. ZW, XY, ZC, CL, GQC, and JD carried out the analysis. ZW, YW, ZY, YG, and ZY wrote the first draft of the manuscript. ZW was the main contributor to the manuscript. GCC, WL, and XX reviewed and revised the manuscript. GCC, WL, and XX oversaw the implementation of the entire research program. All authors reviewed and approved the final submitted version.

## Conflict of Interest

The authors declare that the research was conducted in the absence of any commercial or financial relationships that could be construed as a potential conflict of interest.

## Publisher’s Note

All claims expressed in this article are solely those of the authors and do not necessarily represent those of their affiliated organizations, or those of the publisher, the editors and the reviewers. Any product that may be evaluated in this article, or claim that may be made by its manufacturer, is not guaranteed or endorsed by the publisher.
